# Inflammation, oxidative stress and mitochondrial dysfunction in the progression of type II diabetes mellitus with coexisting hypertension

**DOI:** 10.3389/fendo.2023.1173402

**Published:** 2023-06-13

**Authors:** Hibba Yousef, Ahsan H. Khandoker, Samuel F. Feng, Charlotte Helf, Herbert F. Jelinek

**Affiliations:** ^1^ Department of Biomedical Engineering, Khalifa University, Abu Dhabi, United Arab Emirates; ^2^ Healthcare Engineering Innovation Center, Khalifa University, Abu Dhabi, United Arab Emirates; ^3^ Department of Science and Engineering, Sorbonne University Abu Dhabi, Abu Dhabi, United Arab Emirates; ^4^ Dermatology, Venereology and Allergology, University Hospital Schleswig-Holstein, Schleswig-Holstein, Germany; ^5^ Biotechnology Center, Khalifa University, Abu Dhabi, United Arab Emirates

**Keywords:** type II diabetes, hypertension, oxidative stress, inflammation, mitochondrial dysfunction, prediabetes

## Abstract

**Introduction:**

Type II diabetes mellitus (T2DM) is a metabolic disorder that poses a serious health concern worldwide due to its rising prevalence. Hypertension (HT) is a frequent comorbidity of T2DM, with the co-occurrence of both conditions increasing the risk of diabetes-associated complications. Inflammation and oxidative stress (OS) have been identified as leading factors in the development and progression of both T2DM and HT. However, OS and inflammation processes associated with these two comorbidities are not fully understood. This study aimed to explore changes in the levels of plasma and urinary inflammatory and OS biomarkers, along with mitochondrial OS biomarkers connected to mitochondrial dysfunction (MitD). These markers may provide a more comprehensive perspective associated with disease progression from no diabetes, and prediabetes, to T2DM coexisting with HT in a cohort of patients attending a diabetes health clinic in Australia.

**Methods:**

Three-hundred and eighty-four participants were divided into four groups according to disease status: 210 healthy controls, 55 prediabetic patients, 32 T2DM, and 87 patients with T2DM and HT (T2DM+HT). Kruskal-Wallis and χ2 tests were conducted between the four groups to detect significant differences for numerical and categorical variables, respectively.

**Results and discussion:**

For the transition from prediabetes to T2DM, interleukin-10 (IL-10), C-reactive protein (CRP), 8-hydroxy-2’-deoxyguanosine (8-OHdG), humanin (HN), and p66^Shc^ were the most discriminatory biomarkers, generally displaying elevated levels of inflammation and OS in T2DM, in addition to disrupted mitochondrial function as revealed by p66^Shc^ and HN. Disease progression from T2DM to T2DM+HT indicated lower levels of inflammation and OS as revealed through IL-10, interleukin-6 (IL-6), interleukin-1β (IL-1β), 8-OHdG and oxidized glutathione (GSSG) levels, most likely due to antihypertensive medication use in the T2DM +HT patient group. The results also indicated better mitochondrial function in this group as shown through higher HN and lower p66^Shc^ levels, which can also be attributed to medication use. However, monocyte chemoattractant protein-1 (MCP-1) levels appeared to be independent of medication, providing an effective biomarker even in the presence of medication use. The results of this study suggest that a more comprehensive review of inflammation and OS biomarkers is more effective in discriminating between the stages of T2DM progression in the presence or absence of HT. Our results further indicate the usefulness of medication use, especially with respect to the known involvement of inflammation and OS in disease progression, highlighting specific biomarkers during disease progression and therefore allowing a more targeted individualized treatment plan.

## 1 Introduction

Type II diabetes mellitus (T2DM) is one of the leading causes of disability and premature death globally (1), with numbers expected to rise to 700.2 million by 2045 (2). Prediabetes, the condition preceding T2DM, is expected to reach a global prevalence of 8.3% by the same year (3). Prediabetes is the intermediate state between normoglycemia and overt T2DM (4). While this condition is often asymptomatic (5), pathological processes and hyperglycemia-associated complications can already be observed (6).

T2DM is now regarded as a redox and inflammatory disease (7, 8). Oxidative stress (OS) is not only a result of insulin resistance, the primary characteristic of T2DM, but also a factor in its development and exacerbation. Pancreatic β cells have a higher vulnerability to reactive oxygen species ROS as a result of their weaker antioxidant defense system, leading to their dysfunction and cell death (9). Furthermore, OS interferes with β cell development and function, decreasing the quantity and quality of secreted insulin (10, 11). OS can also compromise insulin function by interference with insulin signaling pathways. The combined impairment of insulin secretion and function is pivotal in the progression of hyperglycemia as is the case in prediabetes to diabetes (10).

Due to the high reactivity and limited half-life of ROS, they are inefficient as biomarkers for OS. Alternatively, adverse effects of ROS and resultant damage to lipids, protein and nucleic acid are more effective estimates of OS (9). An example of lipid peroxidation biomarkers is 8-isoprostane, the result of arachidonic acid peroxidation, which is a reliable biomarker due to its high stability (12). Detrimental effects to nucleic acids can be observed through levels of 8- hydroxy-deoxyguanosine (8-OHdG), a known biomarker of ROS-induced DNA damage (9).

Other than oxidative damage, biomarkers of the antioxidant defense system can also be used as a measure of OS. In erythrocytes, reduced glutathione (GSH) is a non-enzymatic antioxidant, detoxifying ROS that enter the bloodstream. As part of GSH function as an electron scavenger, glutathione peroxidase 1 oxidizes GSH to glutathione disulfide (GSSG). Hyperglycemia reduces the amount of GSH present by hindering the transport of its precursor, cysteine, across the erythrocyte membrane due its increased rigidity (13). Hence, measures of GSH and GSSG are useful in determining oxidative stress status.

Given that the mitochondrial electron transport chain is the principal generator of ROS, mitochondrial dysfunction (MitD) has also been implicated in insulin resistance and shortage (14). Furthermore, MitD is the most likely culprit behind lipotoxicity, referring to excess fat accumulation in the liver and skeletal muscles. Impaired mitochondrial oxidative capacity, found in T2DM patients, hinders metabolism of fatty acids, resulting in the buildup of lipotoxic lipids in the liver and skeletal muscles. Particularly, ceramide and diacylglycerol deposition has been associated with insulin resistance as a result of phosphorylation of essential intermediates in insulin signaling pathways (15, 16).

Biomarkers of MitD have been associated with T2DM, specifically the mitochondrial-derived peptide (MDP) humanin (HN), which is expressed from the short open reading frame of the mitochondrial genome (17). Studies on diabetic rodents have demonstrated that HN plays a role in insulin sensitivity and metabolism by improving mitochondrial function and responding to OS (18, 19). Another marker of MitD, a nuclear-derived molecule, is p66^Shc^, which is involved in the generation of mitochondrial ROS (20). The ShcA locus on chromosome 1 encodes three isoforms, of which p66^Shc^ is the largest (21). P66shc utilizes electrons from the mitochondrial electron transport chain to oxidize cytochrome c, thereby generating ROS (22).

In diabetes mellitus, inflammatory factors are mainly produced by visceral white adipose tissue. Various mechanisms such as hypoxia and macrophage infiltration promote the secretion of cytokines and chemokines (23), all of which are prompted by hyperglycemia (24). Inflammatory factors, in addition to OS, play a significant role in the development of diabetes by promoting insulin resistance through the alteration of β-cell function and interference with insulin signaling (24). Given the crucial role of interleukins in mediating inflammation, through both proinflammatory and anti-inflammatory properties, various interleukins have been investigated in T2DM patients. Interleukin-6 (IL-6) (25), interleukin-1β (IL-1β) (26–28) and interleukin-10 (IL-10) (29) have all been hypothesized to have multifunctional roles in the development of T2DM.

Hypertension (HT) is a frequent comorbidity of diabetes, with the coexistence of both conditions increasing the risk of microvascular and macrovascular complications (30, 31), including a threefold increased risk of cardiovascular disease (CVD) (32). The two conditions coincide often as a result of shared risk factors, particularly insulin resistance (33), with an estimated 75% of T2DM patients developing HT (32). OS and inflammatory processes in diabetes lead to vascular remodeling, which is the alteration of vessel structure, starting with endothelial dysfunction (34). OS and inflammation contribute to the development of endothelial dysfunction through the inactivation of nitric oxide, which increases the risk of HT, primarily through the renin-angiotensin-aldosterone system (35).

The main aim of this study was to identify distinguishing biomarkers of inflammation, OS, and MitD between the different phases of T2DM progression from controls, to prediabetes to T2DM and coexisting T2DM with HT. Investigating these biomarkers can assist in elucidating pathological mechanisms that are not yet fully understood, particularly concerning the transition from prediabetes to T2DM and the development of HT in established T2DM. The current study highlights the differences between T2DM and coexisting T2DM and HT in terms of inflammation, OS and MitD as these are largely understudied and underreported in the literature. Furthermore, the inclusion of novel mitochondrial biomarkers adds additional insight for new, potential therapeutic targets.

## 2 Materials and methods

### 2.1 Dataset, participants and inclusion/exclusion criteria

Participants in this study were recruited from a diabetes screening clinic (DiabHealth) in Albury, Australia, and approved by the Charles Sturt University Ethics in Human Research Committee (Ethics Approval number: 2006/042). Exclusion criteria included those presenting with CVD (coronary artery disease etc.), kidney disease, or acute inflammation. The remaining 384 participants were divided according to disease status into the following four groups: 210 controls (no T2DM or HT), 55 prediabetes (no HT, with 5.5< fasting blood glucose levels (BGL)< 7 mmol/L), 32 T2DM and 87 T2DM+ HT (coexisting T2DM and HT).

A positive diagnosis of T2DM includes any of the following criteria according to the American Diabetes Association (ADA) (36). Presenting with a fasting BGL ≥7 mmol/L, reporting a prior diagnosis, or on glucose-lowering medication. A diagnosis of prediabetes was also established according to ADA guidelines, with a fasting BGL between 5.6 and 6.9 mmol/L. HT is defined by the Australian Heart Foundation criteria (37) as a systolic blood pressure ≥ 140 mmHg and/or a diastolic blood pressure ≥ 90 mmHg on two separate occasions, reporting a prior diagnosis or on antihypertensive medication.

The data collected in this study was part of clinical practice rather than performed in a controlled setting, therefore, patients on hypoglycemic medications, antihypertensive medications including calcium channel blockers, beta blockers, angiotensin-converting enzyme (ACE) receptor blockers and ACE inhibitors, in addition to preventative statin use were not excluded.

### 2.2 Biomarker selection 

For biomarkers of OS, 8-OHdG, 8-isoprostane and GSH/GSSG were selected. 8-OHdG and 8-isoprostane were selected as they are stable biomarkers of DNA oxidative damage (9) and arachidonic acid peroxidation (12), respectively. GSH and GSSG were selected as they represent the efficiency of the antioxidant defense system (13).

For biomarkers of inflammation, monocyte chemoattractant protein-1 (MCP-1), insulin-like growth factor-1 (IGF-1), C-reactive protein (CRP), and IL-6, IL-10 and IL-1β were selected. The traditional inflammatory marker C-reactive protein is not specific for T2DM, but may be combined with other inflammatory markers to enhance specificity (38), hence the inclusion of the remaining biomarkers. MCP-1 was selected as it is a highly studied chemokine with links to metabolic syndrome (39). IGF-1 was also included as it is associated with the incidence of T2DM due to its role in regulation of insulin sensitivity (40). Finally, IL-6, IL-10 and IL-1β were selected due to their pleiotropic roles in T2DM and its complications (25–29).

As for MitD, the biomarkers HN and p66^Shc^ were selected. HN improves mitochondrial function through antiapoptotic activity (41), while p66^Shc^ stimulates apoptosis and increases mitochondrial ROS production (42). Both biomarkers have been linked to T2DM pathology (18–20).

### 2.3 Biomarker collection methods

Fasting BGL of participants was determined through finger prick point of care testing (Accu-Chek^®^ system; Hoffman-La Roche Ltd., Basel, Switzerland). HbA1c, triglycerides, and cholesterol levels were provided by an accredited pathology laboratory as detailed in (32). Brachial artery blood pressure was measured with a Welsh-Allyn BP recorder in a supine position following a five-minute rest and repeated two days later if elevated blood pressure was suspected (32).

Levels of inflammatory markers IL-6, IL-10, IL-1β, monocyte chemoattractant protein-1 (MCP-1), and insulin-like growth factor-1 (IGF-1) were determined using ELISA from urine samples utilizing ELISA provided by Elisakit.com™ (Jomar Pty Ltd, Melbourne, Australia).The sandwich ELISA method was deployed, with the addition of HRP-conjugated streptavidin and AChE-substrate (acetylcholine and 5,5′-dithio-bis-2-nitrobenzoic acid) for the monitoring of color development according to assay protocol (32). Serum CRP levels were provided by Dorevitch Pathology Laboratory, Albury, NSW.

OS and mitochondrial biomarker levels were measured from urinary samples. GSH and GSSG were determined by the Glutathione Colorimetric Detection Kit (Thermo Fisher Scientific, USA). Samples are divided to determine both GSH and GSSG. GSSG is determined by using 2-vinylpyridine to block any free GSH in the sample. Any samples that have not been treated with 2-vinylpyridine will yield total GSH. The Free GSH concentration in the sample is calculated from the difference between the Total GSH determined and the GSH generated from oxidized glutathione for the 2-vinylpyridine-treated samples. The concentration of GSH was determined by calorimetric measures at 405 nm (43).

HN levels were measured via ELISA through plasma samples using the Humanin ELISA Analysis method (Lot No. K11064644) suggested by Elisakit.com (Adelaide, Australia) (44).

Urinary samples were utilized for measurement of 8-OHdG and 8-isoprostane levels. Using 8-hydroxy-2-deoxy Guano-sine EIA Kit (Cayman Chemical, USA), 8-OHdG levels were determined through competitive assay binding and AChE-substrate for color development monitoring (43).

8-isoprostane was measured with Isoprostane ELISA Kit (Northwest, USA), also utilizing competitive assay binding and color monitoring with horseradish peroxidase (43).

Finally, p66^Shc^ levels were determined using a Human SHC-Transforming Protein 1 ELISA kit (CUSA- BIO; Flarebio Biotech LLC) from urinary samples following the avidin-biotin complex method for color reaction (20).

Spectrophotometry for ELISA was conducted according to supplier specifications. A Thermo Scientific Multiskan FC™ (Thermo Fisher Scientific, Waltham, MA, USA) and 4-parameter logistic curve fit software were utilized for ELISA measurements and data analysis, respectively.

### 2.4 Data imputation and statistical analysis

Missing values were present throughout the dataset for various biomarkers as described in (45), which were imputed as detailed in (46) and (47). Continuous variables were confirmed to have a non-normal distribution using Shapiro-Wilk tests, and were thus expressed as median (25th percentile, 75th percentile). Kruskal-Wallis tests were used to detect significant differences between the four groups, followed by Dunn’s *post hoc* test with Bonferroni correction. For categorical variables, χ2 tests were used to compare all four groups, followed by paired χ2 tests with Bonferroni adjustment if significant differences were detected. Significance was set at *p*< 0.05. All statistical tests were performed using RStudio (1.4.1717).

Given that this study was performed on previously collected data, sample size calculations were not performed prior to statistical testing. However, for null results, where the null hypothesis, H_0,_was not rejected, sample size estimations were performed using previously reported results to estimate whether the study was underpowered, and to provide guidance for future studies on reasonable sample sizes to obtain significant results (48). The following formula was used for sample size calculation, where σ - pooled standard deviation, d - difference of means of 2 groups, Z_1-β_ - 0.84 for power 0.80 and Z_α/2_ -1.96 for alpha 0.05 (49):


N=2(Z1−β+Zα/2)c^2(σ)^2d^2

## 3 Results

Number of participants, general clinical characteristics, and biochemistry data are displayed in **Tables 1**, **2**. Participants in the control group had a significantly lower age. Statin use was significantly higher in the T2DM-HT group (48%) than in all other groups, and significantly higher in T2DM (21%) than the prediabetes and control groups. This was reflected in the lipid profiles, as LDL was significantly lower in T2DM and T2DM-HT. Triglycerides were significantly lower in the control and prediabetes groups.

**Table 1:** Clinical characteristics of the four participant groups.

Characteristic	Controls, n= 210	Prediabetes, n= 55	T2DM, n= 32	T2DM+HT, n= 87	*p*-value^1^
Categorical	(%)	(%)	(%)	(%)	χ^2^
Sex (F)	119/210 (57%)	32/55 (58%)	21/32 (66%)	48/87 (55%)	>0.9
Alcohol	26/210 (12%)	9/55 (16%)	2/32 (6.2%)	12/87 (14%)	0.6
Smoking	14/210 (6.7%)	5/55 (9.1%)	3/32 (9.4%)	7/87 (8.0%)	>0.9
DM-Meds	0	0	20/32 (62%)	67/87 (77%)	<0.001 ^b,c,d,e^
Antihypertensives	0	0	0	66/87 (76%)	<0.001 ^c,e,f^
Statin Use	4/210 (1.9%)	0%	7/32 (22%)	42/87 (48%)	<0.001 ^b,c,d,e,f^
NSAID Use	0	0	0	0	_
Aspirin Use	0	0	0	0	_
Numerical	Median (Q1-Q3)	Median (Q1-Q3)	Median (Q1-Q3)	Median (Q1-Q3)	Kruskal-Wallis
Age (Years)	52 (45 62)	57 (50,68)	55(49-65)	61(57,70)	<0.001 a^,c^
BMI (Kg/m^2^)	25.7 (23.3, 29.1)	28.1 (24.5, 30.4)	29.2 (23.8, 34.1)	29.4 (26.0, 34.5)	<0.001 ^b,c^
Lying-SBP (mmHg)	120 (113, 128)	126 (116, 132)	128 (118, 132)	140 (130, 152)	<0.001 ^c,e,f^
Lying-DBP (mmHg)	77 (70, 82)	80 (70, 85)	78 (70, 84)	80 (74, 88)	0.01 ^c^
Numerical			Median (Q1-Q3)	Median (Q1-Q3)	Wilcoxon Rank Sum
Duration of diabetes (years)			2.0 (1.0,4.2)	4.0 (1.0, 9.5)	0.060

DM, Diabetes mellitus; NSAID, Non-steroidal anti-inflammatory drugs; SBP, Systolic blood pressure; DBP, Diastolic blood pressure.

^a^ Significant difference between control and prediabetes group (p<0.05); ^b^ significant difference between control and T2DM group (p<0.05); ^c^ significant difference between control and T2DM+HT group (p<0.05); ^d^ significant difference between prediabetes and T2DM group; ^e^ significant difference between prediabetes and T2DM+HT group; ^f^ significant difference between T2DM and T2DM+HT group. -, Not applicable.

**Table 2:** Lipid profile and glycemic measurements of the four participant groups.

Characteristic	Controls, n= 210	Prediabetes, n= 55	T2DM, n= 32	T2DM+HT, n= 87	*p*-value^1^
Numerical	Median (Q1-Q3)	Median (Q1-Q3)	Median (Q1-Q3)	Median (Q1-Q3)	Kruskal-Wallis
BGL (mmol/L)	4.80 (4.43, 5.10)	5.90 (5.70, 6.25)	6.65 (5.93, 9.25)	7.50 (5.95, 9.60)	<0.001 a^,b,c^
HbA1c (%)	5.65 (5.40, 5.90)	5.80 (5.70, 6.10)	7.40 (6.30, 8.20)	7.60 (6.60, 8.15)	<0.001 a^,b,c,d,e^
HDL (mmol/L)	1.30 (1.10, 1.40)	1.50 (1.20, 1.70)	1.20 (1.00, 1.52)	1.30 (1.10, 1.50)	0.006 ^a,d,e^
LDL (mmol/L)	3.10 (2.80, 3.40)	3.00 (2.50, 3.50)	2.70 (1.90, 2.82)	2.70 (2.10, 3.30)	<0.001 ^b,c,d^
Triglycerides (mmol/L)	1.10 (0.90, 1.40)	1.00 (0.80, 1.30)	1.80 (1.23, 3.30)	2.00 (1.50, 2.20)	<0.001 ^b,c,d,e^
TC (mmol/L)	4.95 (4.50, 5.57)	5.00 (4.70, 5.50)	4.50 (3.90, 5.03)	4.60 (4.10, 5.50)	0.001 ^b,c,d^

BGL, Fasting blood glucose levels; HDL, High density lipoprotein; LDL, Low density lipoprotein; TC, Total cholesterol.

^a^ Significant difference between control and prediabetes group (p<0.05); ^b^ significant difference between control and T2DM group (p<0.05); ^c^ significant difference between control and T2DM+HT group (p<0.05); ^d^ significant difference between prediabetes and T2DM group; ^e^ significant difference between prediabetes and T2DM+HT group; ^f^ significant difference between T2DM and T2DM+HT group.

Regarding antihypertensive medication use, 26% (23/87) of patients with DM-HT were on angiotensin II receptor blockers (ARB), 25% (22/87) on angiotensin converting enzyme inhibitors, 10% (9/87) on calcium channel blockers, on 15% (13/87) β-blockers and 20% (17/87) on diuretics.

BMI was only significantly different between the control group, T2DM, and T2DM-HT groups. Furthermore, as expected, BGL was significantly higher in prediabetes, T2DM, and T2DM-HT groups than in the control group, though no differences were observed between the prediabetes, T2DM, and T2DM-HT groups. HbA1c was significantly higher in T2DM and T2DM-HT groups than in control and prediabetes groups. As expected, lying-SBP and DBP were significantly higher for the T2DM-HT group. In addition, patients in the T2DM-HT group had a longer duration of diabetes than in the T2DM group, with statistical significance almost achieved (p = 0.06).

Values for the OS, inflammation, and MitD biomarkers are shown in **Table 3**. For inflammatory biomarkers, IL-1β was significantly increased in prediabetes and T2DM compared to controls (p< 0.001). Levels then became comparable to controls again in the T2DM-HT group (p = 0.5). Similarly, IL-10 levels were significantly lower in T2DM than in controls (p< 0.001) and prediabetes (p< 0.001) but increased in the T2DM-HT group (p = 0.02). IL-6 and CRP displayed similar patterns to IL-1β. However, MCP-1 showed a sequential increase from the control group to T2DM-HT, though this increase was not significant for all intergroup group comparisons. As for IGF-1, prediabetes, T2DM and T2DM-HT were all significantly lower than the control group, but no significant differences were present between the three groups.

**Table 3:** Biomarker levels for the four participant groups.

Characteristic	Controls, n= 210	Prediabetes, n= 55	T2DM, n= 32	T2DM+HT, n= 87	*p*-value^1^
Numerical	Median (Q1-Q3)	Median (Q1-Q3)	Median (Q1-Q3)	Median (Q1-Q3)	Kruskal-Wallis
IL-6 (pg/mL)	16 (12, 19)	15 (12, 21)	39 (3, 73)	12 (6, 34)	0.092 ^b,f^
IL-10 (pg/mL)	35 (22, 99)	30 (27, 44)	18 (18, 19)	29 (16, 65)	<0.001 ^b,c,d,f^
IL-1β (pg/mL)	5 (3, 9)	8 (5, 24)	11 (10, 15)	5 (3, 11)	<0.001 a^,b,f^
MCP-1 (pg/mL)	205 (182, 221)	205 (187, 214)	225 (184, 281)	228 (195, 241)	<0.001 ^b,c,e^
IGF-1 (pg/mL)	236 (161, 375)	180 (69, 225)	112 (90, 122)	148 (86, 193)	<0.001 a^,b,c^
CRP (mg/mL)	1.40 (1.00, 1.80)	2.00 (1.60, 2.30)	5.50 (4.80, 8.00)	2.20 (2.00, 3.05)	<0.001 a^,b,c,d,f^
GSH (μM)	1,584 (1,524, 1,720)	1,629 (1,388, 1,827)	1,740 (1,666, 1,740)	1,314 (1,299, 1,682)	<0.001 ^c,e,f^
GSSG (μM)	270 (243, 318)	345 (249, 358)	351 (275, 412)	261 (215, 357)	<0.001 a^,b,e,f^
GSH/GSSG	5.85 (5.01, 6.57)	4.81 (3.95, 7.13)	4.66 (4.23, 5.74)	4.97 (4.23, 7.38)	<0.001^b,c^
8-OHdG (ng/mL)	143 (103, 212)	153 (153, 204)	110 (83, 144)	90 (49, 114)	<0.001 ^b,c,d,e^
8-isoprostane (ng/mL)	1.3 (0.7, 2.9)	1.0 (0.8, 2.0)	1.4 (0.7, 19.1)	1.2 (0.9, 1.9)	0.2
Humanin (pg/mL)	212 (167, 239)	209 (115, 274)	151 (101, 154)	248 (198, 272)	<0.001 ^b,c,d,e,f^
p66-Shc (pg/mL)	46 (38, 56)	39 (35, 50)	52 (51, 67)	39 (34, 56)	<0.001 a^,b,d,f^

8-OHdG, 8-hydroxydeoxyguanosine; GSH, Reduced glutathione; GSSG, Oxidized glutathione; HN, Humanin; IL-6, Interleukin 6; IL-1β, Interleukin 1 beta; IL-10, Interleukin 10; MCP-1, Monocyte chemoattractant protein-1; IGF-1, Insulin-like growth factor-1; CRP, C-reactive protein; C5a, Complement component 5a.

Results of Dunn’s post hoc testing: ^a^ Significant difference between control and prediabetes group (p<0.05); ^b^ significant difference between control and T2DM group (p<0.05); ^c^ significant difference between control and T2DM+HT group (p<0.05); ^d^ significant difference between prediabetes and T2DM group; ^e^ significant difference between prediabetes and T2DM+HT group; ^f^ significant difference between T2DM and T2DM+HT group.

In terms of OS, GSH initially increased, almost achieving statistical significance, from controls to T2DM (p< 0.001), followed by a significant decrease when compared to coexisting T2DM-HT (p< 0.001). GSSG displayed a similar sequence of increase moving from controls to T2DM (p< 0.01), after which a significant decrease occurred in T2DM-HT (p< 0.001). 8-isoprostane did not successfully discriminate between groups. However, 8-OHdG was lower in T2DM and T2DM-HT than in prediabetes (p< 0.001) and controls (p< 0.05, p< 0.001).

For biomarkers of MitD, p66shc, values showed a significant decrease (p = 0.02) moving from controls to prediabetes, after which a significant increase was observed in T2DM (p< 0.001), followed by a significant decrease again when moving to T2DM-HT (p< 0.001). Whereas HN displayed a sequential decrease from controls to prediabetes (p = 1.0), and from prediabetes to T2DM (p< 0.001), followed by a significant increase in value for T2DM-HT (p< 0.001).

## 4 Discussion

The main aim of this study was to investigate differences in biomarker levels of OS, inflammation and MitD between phases of T2DM development. Despite the increased complications in patients with coexisting T2DM and HT (50), this area of study remains underdeveloped, and exact mechanisms distinguishing normotensive and hypertensive individuals with T2DM remain elusive.

Participants in this study were divided into four groups according to disease status, including controls, prediabetic patients, normotensive T2DM patients and those with coexisting T2DM and HT. Given that the data utilized comprised of patients visiting a routine diabetes screening clinic, unequal numbers of participants were found in each of the four groups. About 16% of adults above the age of 25 have prediabetes (51) and around 15.5% aged 65-69 have T2DM in Australia (52). Furthermore, as previously discussed, approximately 25% of T2DM are normotensive. Hence, differences in participant group sizes are to be expected.

Differences in medication use were present between groups, which most likely impacted many of the biomarker levels observed in this study. One example is the lower levels of LDL in T2DM and T2DM-HT groups, which was most likely as a result of a higher proportion of statin use in these groups. Statin therapy lowers LDL by increasing the rate of its uptake by hepatic cells, effective through increasing the density of LDL receptors on hepatic cell surfaces and reducing intrahepatic cholesterol content (53).

The findings in this study displayed a general trend of increased levels of inflammation moving from controls towards prediabetes, a further increase when transitioning to T2DM, followed by suppressed inflammation in coexisting disease (T2DM-HT).

IL-6 levels slightly decreased moving from controls to prediabetes, then exhibited a rise in T2DM. This was in partial agreement with previous results, where IL-6 levels were higher in both prediabetes and T2DM than controls (54). IL-6 has two distinct signaling pathways, classic and trans-signaling, each exhibiting opposite effects on inflammation and glucose metabolism depending on the activated pathway (25). The drop and subsequent rise in IL-6 levels may allude to transitioning functions of IL-6 during the progress of disease.

In agreement with previous work regarding inflammatory biomarkers associated with T2DM, our results show significantly higher IL-6, CRP (43), and IL1-β (38), and significantly lower IL-10 levels and IGF-1 (55) in T2DM in comparison to controls. CRP and IL-10 were also discriminatory between prediabetes and T2DM, indicating their potential efficiency as biomarkers alongside BGL for the transition from prediabetes to T2DM disease status.

IL-10 and IGF-1 decreased sequentially from controls to T2DM. Previous studies have revealed both decreased and increased levels of IL-10, decreased expression of IL-10 receptors and defective IL-10 function in T2DM patients (29, 55). The anti-inflammatory IL-10 imparts protection against insulin resistance through its modulation of cytokine production by macrophages (55). Though Barry et al. found increased levels with reduced function of IL-10 in T2DM patients (29), our results are in agreement with Abhilasha et al. (55), where lower levels of IL-10 were found. While this finding was not significant, our study revealed lower levels of IL-10 in prediabetes compared to controls, in contrast to results obtained by Wang et al. (56). These results are not contradictory, however, as they represent cytokine levels at a single point in time, and may be at different timelines throughout the development of the disease. Furthermore, the aforementioned finding of impaired IL-10 function may indicate that both a decrease in quality and quantity of IL-10 is involved in T2DM development.

The significant decrease in inflammation for the T2DM+HT group is most likely explained by the use of antihypertensive medication in this group. Previous findings have revealed anti-inflammatory effects of a variety of commonly used antihypertensive medications (57). This effect was demonstrated through increased IGF-1 and IL-10, in addition to reduced levels of IL-6, CRP, and IL1-β moving from T2DM to coexisting T2DM and HT. MCP-1, however, did not exhibit a similar pattern, as elevated levels persisted even in T2DM-HT. This corroborates earlier work by Rabkin et al. (58), where MCP-1 was significantly higher in patients with both T2DM and HT, in whom normal antihypertensive medication use was resumed for the duration of the study. Furthermore, the relationship between MCP-1 and HT has also been established previously given the important role of this chemokine in endothelial dysfunction and vascular remodeling (59, 60). Angiotensin II receptor blockers (ARB) are a class of antihypertensive medication that function by competitively displacing angiotensin II. Angiotensin II has two receptors, AT1R and AT2R. The specific mechanism of action of ARBs is by inhibiting AT1R and allowing angiotensin II binding to AT2R (61). ARBs are beneficial in the treatment of HT not only through lowering blood pressure, but also by ameliorating inflammation through AT1R blockade (62). Interestingly, however, previous findings have indicated the potential role of AT2R in mediating the release of MCP-1 (63), which may explain the sustained elevation of this biomarker in the T2DM-HT group, given that a large proportion of patients were on ARBs. MCP-1 results indicate that MCP-1 may act as a promising biomarker for prediction of HT development in T2DM patients, however, longitudinal studies are necessary to confirm this.

Unlike previous findings (32, 64), 8-isoprostane did not discriminate between the OS status of the four groups in this study, However, Ma et al. found no correlation between F2- isoprostanes and insulin resistance (65), and F2-isoprostanes may also represent a biomarker of intensive metabolism, with higher levels reflecting increased fatty acid oxidation and better metabolic adaptation, through which the risk of T2DM could be lowered (66). Based on previous results (32), an effect size and standard deviation of about 0.85 and 3.3 ng/mL, respectively, of mean 8-isoprostane levels can be expected between groups. Given a power of 80% and at *p*< 0.05, a sample size of around 274 would be required to obtain significant results (sample size = 2(1.96+0.84)^2(3.3)^2(0.85)^2 ) Therefore, a larger cohort is needed to further investigate this relationship, especially given the lower numbers of participants in the prediabetes and T2DM groups.

In contrast, 8-OHdG levels did differ between groups, being lower in T2DM than in controls and prediabetes in contrast to previous results. This may reflect the impact of antidiabetic, antihypertensive medication as well as statins. Hypoglycemic drugs including metformin, sulfonylureas and thiazolidinediones exhibit antioxidant properties through scavenging of ROS, inhibiting advanced glycation end-product formation and upregulating antioxidants (67). In addition, although studies have not proven this effect, it is hypothesized that statin therapy may impact levels of oxidative DNA damage by alleviating dyslipidemia, leading to a reduction in 8-OHdG (68). This is further illustrated by the significantly lower levels of 8-OHdG in the T2DM+HT group, where a significantly higher proportion of participants were on statin therapy. Combined with several antihypertensive medications, including the beta-blockers metoprolol, carvedilol, and bisoprolol, and ACE inhibitors that have been reported to reduce OS (69, 70), our results indicate the effectiveness of these medications in reducing OS. Longer follow-up studies are necessary to confirm whether the decrease in oxidative stress leads to an improvement in patient disease progression.

The present findings also verify previous results (43) showing increased GSH production with T2DM in response to rising OS, which is also reflected through rising GSSG levels. The decreasing levels of GSH moving towards T2DM+HT agree with reported results showing decreased efficiency of GSH production in the presence of coexisting disease (32). Lower levels of OS, reflected in lower GSSG levels, may result from the aforementioned antihypertensive medication effects. Furthermore, recycling of GSSG is not the only method of GSH production, which is also synthesized from precursors glycine and cysteine, both found to be significantly reduced in T2DM (71).

Novel findings in this study included the analysis of mitochondrial biomarkers in the various disease stages. Similar to a previous finding by (44), HN significantly decreased moving from controls towards T2DM. However, the trend was reversed, and levels increased once again in T2DM+HT, becoming significantly higher than in the control group. Given the critical role of HN in lowering OS (72), this may act as a marker for the efficiency of antihypertensive medication therapy in immunomodulation and alleviation of associated MitD.

The role of p66^Shc^ in T2DM is highly inconclusive with conflicting results obtained in mouse models, however, higher levels of p66^Shc^ expression are believed to suppress insulin signaling pathways, induce pancreatic β-cell apoptosis and reduce insulin secretion (73, 74). p66^Shc^ expression is regulated by the sirtuin SIRT1 (75).The significant decrease in prediabetes from controls agreed with previous findings (20), where hyperglycemia initially suppresses p66^Shc^ due to SIRT1 activity. Subsequently, SIRT1 levels may drop in diabetic patients, as supported by previous findings, allowing for elevation of p66^Shc^ levels (76). Once again, a drop in p66^Shc^ levels was observed in the T2DM-HT group, which could also be explained by the aforementioned immunomodulatory effects of antihypertensive medications, especially given the role of p66^Shc^ in producing ROS in the mitochondrial electron transport chain (77).

In terms of HT, our results indicated a significant increase in the DM+HT group in systolic blood pressure, when combined with the findings of the oxidative and inflammatory biomarkers, can guide physicians in choosing appropriate medications to reduce blood pressure in this group. The minor increases in BP for the prediabetes and T2DM groups can be reviewed similarly as a proactive treatment option. Overall, the observed changes in OS and inflammatory markers, when combined, indicate significant differences associated with diabetes progression from no diabetes to DM+HT in a clinical setting where patients are necessarily on medication suggest the usefulness of including additional biomarkers to the patient review.

This study has several limitations. First, the four groups of interest were not similar in size. However, given that this data was collected as part of a diabetes screening effort, differences in number of participants reflect proportions of these groups in the population (78). Another limitation is the lack of information on dietary intake or nutritional supplementation in the dataset. Studies have found that supplements such as vitamin E have antioxidant and anti-inflammatory properties (79, 80), which may have the potential to alter related biomarker levels. Therefore, data collection in future studies should also encompass this information. Finally, the data collected on subjects in this study was part of clinical practice rather than performed in a controlled setting. This precluded homogeneity in medication use between contrasted populations, particularly for patients with coexisting T2DM and HT on antihypertensive medications. Consequentially, biomarkers revealing disease mechanisms may have been masked as a result of medication use. To address this, newly diagnosed patients for analysis prior to the initiation of pharmacotherapy could be recruited. Alternatively, for clinical practice, a realistic model needs to be developed similar to measures for blood pressure where a cut-off has been proposed regardless of comorbidities or medication use.

Prevalence of T2DM and co-existing complications such as HT is still increasing. Current knowledge on disease progression in general has highlighted the role of inflammation and oxidative stress as well as the important role of mitochondria that is compromised in diabetes progression (81). The current study highlights the benefit of measuring these biomarkers, which may lead to more effective treatment outcomes (82–84).

## 5 Conclusion

Several important conclusions can be inferred. First, the study confirmed previous findings of elevated inflammation and OS in prediabetes and T2DM. Second, OS and inflammatory biomarkers may be effective in monitoring the effectiveness of antihypertensive medications. Furthermore, MCP-1 appears to be a reliable biomarker for HT even with the use of medication, and may serve as a good predictive factor in follow-up, longitudinal studies. Novel MitD biomarkers HN and p66^Shc^ show promising potential for future targeted therapy in the prevention and treatment of T2DM. This study is the first to provide measures of inflammation, OS and MitD in clinical practice, that can be used as the basis for determining threshold values in future studies and provide individualized medicine based on these markers.

## Data availability statement

The raw data supporting the conclusions of this article will be made available by the authors, without undue reservation.

## Ethics statement

The studies involving human participants were reviewed and approved by Charles Sturt University Ethics in Human Research Committee (Ethics Approval number: 2006/042). The patients/participants provided their written informed consent to participate in this study.

## Author contributions

HY, AK, SF, HJ designed research idea. HY performed research. HY, AK, SF, CH, HJ analyzed data and results. HY, HJ, AK, SF, CH wrote and edited the paper. All authors contributed to the article and approved the submitted version.
